# Psychotic aura symptoms in familial hemiplegic migraine type 2 (*ATP1A2*)

**DOI:** 10.1007/s10194-012-0462-5

**Published:** 2012-06-05

**Authors:** José Barros, Alexandre Mendes, Ilda Matos, José Pereira-Monteiro

**Affiliations:** 1 Serviço de Neurologia, Hospital de Santo António (HSA), Centro Hospitalar do Porto (CHP), Largo Professor Abel Salazar, 4099-001 Porto, Portugal; 2 Instituto de Ciências Biomédicas Abel Salazar (ICBAS), Universidade do Porto, Porto, Portugal; 3 Serviço de Neurologia, Unidade Local de Saúde do Nordeste (ULSN), Mirandela, Portugal

**Keywords:** ATP1A2 gene, Familial hemiplegic migraine type 2, M731T mutation, Neuropsychological aura, Psychosis, Time travel

## Abstract

**Introduction:**

Neuropsychological symptoms are rare in familial hemiplegic migraine (FHM). There are no reports of psychotic symptoms in FHM type 2 (*ATP1A2*). We examined a family with a FHM phenotype due to a M731T mutation in *ATP1A2*. A 10-year follow-up allowed us to observe complex auras, including psychotic symptoms in two siblings.

**Case report:**

Male, 48 years old, with an aura that included complex illusions with a feeling of time travelling, coincident with other aura features. The aura was regarded as mystical by the patient. Female, 38 years old, with a complex migraine aura, during which she believed she had the ability to time travel and was being followed by lobbyists who wanted to steal this ability from her.

**Discussion:**

FHM type 2 must be included in the list of differential diagnoses of acute psychosis in patients with a previous history of migraine aura.

## Introduction

Familial hemiplegic migraine (FHM) is an autosomal dominant type of migraine with aura (MA). The diagnostic criteria require the presence of a reversible motor deficit associated with at least one other transient neurological symptom and similar episodes in relatives [1]. FHM may be associated with mutations in the genes *CACNA1A* (FHM1), *ATP1A2* (FHM2) and *SCN1A* (FHM3) [1]. Mutations in the *ATP1A2 gene*, on 1q23, which encodes the alpha 2 subunit of the Na^+^,K^+^-ATPase [2], are sometimes associated with complex paroxysmal episodes or permanent neurological signs [3, 4]. Reports of psychotic symptoms in FHM1 are very rare [5–7]. There is no data concerning familial cases caused by *ATP1A2* mutations (FHM2); however, a delusional misidentification syndrome was described in one sporadic case [8].

**Fig. 1:**
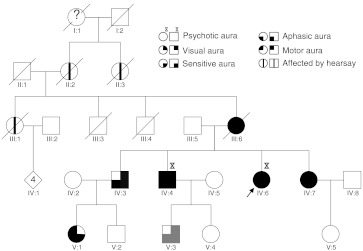
Pedigree structure. Migraine auras are represented by *different symbols* (see above). *Gray*-filled quadrants represent typical aura of an individual without *ATP1A2* mutation. *Arrow* indicate proband

A 10-year follow-up of a three-generation family (Fig. 1) allowed us to document two siblings with illusion of time travelling included in the migraine aura.

## Patients and methods

All members with migraine aura were examined at home in January 2002. Blood samples were collected for genetic study, but patients IV:4, V:1 and V:3 refused testing. In 2007, a M731T mutation in the *ATP1A2* gene was found [9]. Individuals IV:6 and IV:7 were clinically followed carefully. All living members were re-examined in January 2012. At that time, the patients agreed to provide saliva, and the genetic study was completed. An informed consent form was signed by all family members.

## Results

We examined 7 patients (6 still living). The M731T mutation in *ATP1A2* was confirmed in all five patients with hemiplegic aura and in one case of typical aura (IV:3). The mutation was not found in one patient who had a typical aura in childhood (V:3). The detailed main features of mutation carriers are described in Table 1. The age at onset of the aura symptoms ranged from 9 to 15 years old (average = 12.3 years; SD = 2.8 years). Three individuals entered apparent remission at different ages. In contrast, the matriarch, who died from pneumonia at 73, suffered episodes her entire life. The number of episodes throughout life ranged from 2 (V:1) to more than 100 (III:6). The duration of the aura ranged from 15 min to 16 days (IV:7). The clinical spectrum included visions of zigzag lines or scintillating scotoma, numbness, hemiparesis, aphasia and illusions of time travel. The motor aura affected the upper limbs in all patients. The sensitive or motor auras were bilateral in one case and shifted sides in the other five. Aphasia was present in patients with either right- or left-sided paresis. The headache exhibited a variable duration and had a throbbing or tightening character with a moderate intensity in most cases. Several different triggers were reported.

**Table 1:** Main clinical features of family members with *ATP1A2* mutation

Subject/gender/age (years)	Onset, remission, (peak) (years)	Lifetime occurrence (no. episodes)	Aura				Headache				Accompanying symptoms	Precipitating factors
			Duration	Type	Location	Laterality	Duration (h)	Character	Laterality	Intensity		
III:6/F/(73)	15 (20–50)	>100	15 min–1 h	Scintillating scotoma; sensitive; motor; aphasic	F, T, UL, LL, Tr	Side-shifting	72 h	Pressing/tightening	Side-shifting	Moderate	N, V	Menstruation. Pregnancy.
IV:3/M/51	10, 44 (10–16)	>40	15 min	Zigzag lines; sensitive; aphasic.	UL	Side-shifting (90 % right)	6 h	Throbbing	Bilateral	Moderate	N, Pt, Pn	Stress.
IV:4/M/48	16, 45 (26–30)	>20	30–60 min	Scintillating scotoma; sensitive; motor; aphasic; illusion of time-travel.	F, T, UL, LL, Tr	Bilateral (50 %) or Side-shifting (70 % right)	8 h	Throbbing	Side-shifting (70 % right)	Severe	N, V, Pt, Pn Sneezing	Chocolate. Heading.
IV:6/F/38	11 (36–38)	>50	1 h–5 days	Scintillating scotoma; sensitive; motor; aphasic; illusion of time-travel.	F, T, UL, LL, Tr	Side-shifting (80 % left)	24–72 h	Throbbing	Side-shifting (80 % left)	Severe	N, V, Pt, Pn, Dizziness	Menstruation.
IV:7/F/35	9 (33–35)	>30	15 min-16 days	Scintillating scotoma; sensitive; motor; aphasic.	F, T, UL, LL	Side-shifting (80 % left)	30 min	Pressing/tightening	Side-shifting	Moderate	N, V, Pt, Pn,	Menstruation, chocolate, coffee. Hitting a goal post with her head. Immediate puerperium.
V:1/F/19	13, 15	2	15 min	Sensitive; motor; aphasic.	F, T, UL	Side-shifting	30 min	Pressing/tightening	Bilateral	Moderate	N, Pt, Pn	Facial trauma.

*F* face, *T* tongue, *UL* upper limb, *LL* lower limb, *Tr* trunk, *N* nausea, *V* vomiting, *Pt* photophobia, *Pn* phonophobia, *S* somnolence

## Siblings with psychotic aura

IV:4. Male, 48 years old, bricklayer. Between the ages of 20 and 35 years, his auras included a feeling of time travelling. He felt as if he was being sucked into a spiralling tunnel that led to a luminous and serene paradise. After a few minutes in what he described as the afterlife, he was sent back. These episodes were always part of the aura, usually coinciding with aphasia and motor deficit. Between episodes, he made mystical interpretations regarding these phenomena and was proud to be a predestined.

IV:6. Female, 38 years old, office employee. At 37 years-old, while on vacation, she arrived by taxi at her sister’s house, hundreds of miles away from the town where she was supposed to be. She looked frightened and restless, believing she was being followed by telecom operator lobbyists who were competing to provide services for her ability to time travel. She became aggressive against her relatives because she believed they were accomplices of her pursuers. She would brandish a knife, calling it “the key of time”. She was admitted to the psychiatry department; at neurological examination she had dysphasia and right central facial palsy. A 1.5-tesla MRI and EEG were normal. All symptoms disappeared in a 48 h period. She did not comply with the neuroleptics after discharge and continued having rare episodes of hemiplegic migraine, including the feeling of time travelling. She is aware that the migraine is the cause of these symptoms and has not had another psychotic episode.

## Discussion

Psychotic symptoms during the aura of hemiplegic migraine have been described in few cases with FHM1 [6], in a sporadic case linked to *ATP1A2* gene mutation [8], and in a few cases without genetic investigation [5]. Transient or permanent neuropsychiatric features have been rarely associated with *ATP1A2* mutations: confusion [4], behavioural disorders [10], borderline personality [11] and cognitive retardation [3, 11]. This is the first description of psychotic aura symptoms in FHM2, thus, widening its phenotypic range. The complex aura in siblings IV:4 and IV:6 exhibited a common trait, namely, the feeling of time travel. Although the acute expression was dramatic in the female, requiring a short hospitalisation, between attacks she understood that her symptoms were due to the migraine. Conversely, her brother believed these phenomena to be unearthly. Non-genetic factors (low schooling level and a secluded rural setting) might at least partially explain his interpretation.

Initially, this family seemed to present with “standard” FHM, but a long-term follow-up allowed unusual symptoms to emerge. Psychotic symptoms may be under-reported, perhaps because patients feel embarrassed to share this type of symptom with the doctor. This prolonged follow-up gives patients and families a more accurate perception and record of the symptoms.

We can speculate about the anatomical location of these symptoms by analogy. There is evidence of a neural network involved in mental time travel, consisting of the medial prefrontal, medial temporal, medial parietal, lateral parietal-occipital and lateral temporal regions [12]. Accepting that the cortex in these regions may be involved via cortical spreading depression is not difficult. However, the basis of voluntary mental time travel may differ from that of illusionary time travel.

The prognosis of FHM tends to be good; however, establishing an individual accurate prognosis may be difficult. The variability of the natural history and prognosis as a function of different *ATP1A2* mutations are well known. Furthermore, individuals with the same mutation may also have very different features, as presented in this family. Two siblings had the illusion of time travelling, but the other family members absolutely denied experiencing such a symptom, even when directly questioned in private. The motor aura lasted more than 1 h in only 2 episodes, but the duration of those 2 was very long: 5 days (IV:6) and 16 days (IV:7). The classical FHM regression in old age was contradicted in the case of the matriarch (III:6); conversely, her granddaughter (V:1) exhibited only two episodes and entered apparent remission at the beginning of adolescence. These capricious and unequal syndromic profiles suggest the existence of powerful endogenous or environmental factors that trigger, modulate or inhibit the genetic background.

Finally, we recommend that FHM2 must be included in the list of differential diagnoses of acute psychosis in patients with previous history of migraine aura.
